# Molecular engineering of a minimal E-cadherin inhibitor protein derived from *Clostridium botulinum* hemagglutinin

**DOI:** 10.1016/j.jbc.2023.102944

**Published:** 2023-01-25

**Authors:** Sho Amatsu, Takuhiro Matsumura, Masahiko Zuka, Yukako Fujinaga

**Affiliations:** 1 Department of Bacteriology, Graduate School of Medical Sciences, Kanazawa University, Ishikawa, Japan; 2 Department of Forensic Medicine and Pathology, Graduate School of Medical Sciences, Kanazawa University, Ishikawa, Japan

**Keywords:** E-cadherin inhibitor, cell–cell adhesion, *Clostridium botulinum*, hemagglutinin, molecular engineering, epithelial barrier disruption, iPSC culture system, aa, amino acid, AJs, adherens junctions, BoNT, botulinum neurotoxin, HA, hemagglutinin, K_D_, dissociation constant, K_off_, dissociation rate constant, K_on_, association rate constant, sc, single-chain, TER, transepithelial electrical resistance

## Abstract

Hemagglutinin (HA), a nontoxic component of the botulinum neurotoxin (BoNT) complex, binds to E-cadherin and inhibits E-cadherin-mediated cell–cell adhesion. HA is a 470 kDa protein complex comprising six HA1, three HA2, and three HA3 subcomponents. Thus, to prepare recombinant full-length HA *in vitro*, it is necessary to reconstitute the macromolecular complex from purified HA subcomponents, which involves multiple purification steps. In this study, we developed NanoHA, a minimal E-cadherin inhibitor protein derived from *Clostridium botulinum* HA with a simple purification strategy needed for production. NanoHA, containing HA2 and a truncated mutant of HA3 (amino acids 380–626; termed as HA3^mini^), is a 47 kDa single polypeptide (one-tenth the molecular weight of full-length HA, 470 kDa) engineered with three types of modifications: (i) a short linker sequence between the C terminus of HA2 and N terminus of HA3; (ii) a chimeric complex composed of HA2 derived from the serotype C BoNT complex and HA3^mini^ from the serotype B BoNT complex; and (iii) three amino acid substitutions from hydrophobic to hydrophilic residues on the protein surface. We demonstrated that NanoHA inhibits E-cadherin-mediated cell–cell adhesion of epithelial cells (*e.g.*, Caco-2 and Madin–Darby canine kidney cells) and disrupts their epithelial barrier. Finally, unlike full-length HA, NanoHA can be transported from the basolateral side to adherens junctions *via* passive diffusion. Overall, these results indicate that the rational design of NanoHA provides a minimal E-cadherin inhibitor with a wide variety of applications as a lead molecule and for further molecular engineering.

*Clostridium botulinum* produces the botulinum neurotoxin (BoNT) complex, one of the most potent natural toxins causing botulism (1). The BoNT complex comprises neurotoxin (BoNT), nontoxic nonhemagglutinin, and hemagglutinin (HA) (2). BoNTs are traditionally classified into seven serotypes (A–G) (1). HA is a nontoxic 470 kDa protein complex comprising six HA1, three HA2, and three HA3 subcomponents (3). HA has at least two biological activities that promote intestinal absorption of BoNTs: carbohydrate-binding activity (4, 5, 6, 7, 8, 9, 10, 11) and E-cadherin-binding activity (12, 13, 14). HAs derived from the serotype A BoNT complex (HA/A) and serotype B BoNT complex (HA/B) bind to the EC1–2 domain of E-cadherin, inhibiting its dimerization and causing epithelial barrier disruption (12, 13).

E-cadherin is an important cell-adhesion molecule that mediates cell–cell adhesion at adherens junctions (AJs) in epithelial cells (15, 16). AJs are located beneath tight junctions, which prevent diffusion of molecules between apical and basolateral membranes (17). Type I classic cadherins, including E-cadherin, contain five extracellular domains (1–5), a single transmembrane domain, and an intracellular domain. The extracellular 1–2 domains of type I classic cadherins bind another cadherin to form transdimer and cisdimer in a calcium ion–dependent manner. E-cadherin-mediated cell–cell adhesion plays a pivotal role in the development and maintenance of tissue organization. E-cadherin-mediated cell contacts are known to inhibit cell proliferation through the Hippo signaling pathway (18). HA/A and HA/B inhibit E-cadherin-mediated cell contacts (12, 13, 19) and promote the proliferation of Caco-2 cells (human colon carcinoma–derived epithelial cell line), T84 cells (human colon carcinoma–derived epithelial cell line), and Madin–Darby canine kidney type I (MDCK-I) cells (canine kidney epithelial cell line) (19). Furthermore, HA disperses human-induced pluripotent stem (iPS) cell aggregates in 3D suspension culture, resulting in a larger number of live cells, higher cell density, and higher-fold expansion than those of cells treated with conventional digestive enzymes (20). Therefore, human iPS cell culture systems with HA facilitate simple and robust maintenance of undifferentiated cells (20, 21, 22).

HA forms a heterododecameric complex that adopts a large triskelion-shaped structure (Fig. 1*A*) (3). In a previous study, the recombinant HA/B complex was reconstituted *in vitro*, by mixing the recombinant proteins of HA1, HA2, and HA3 and incubating at 37 °C for 3 h (3). Thus, three protein expression systems (HA1, HA2, and HA3) and four purification steps (three HA subcomponents and *in vitro* reconstitution) are required to obtain the HA/B complex.

**Figure 1:**
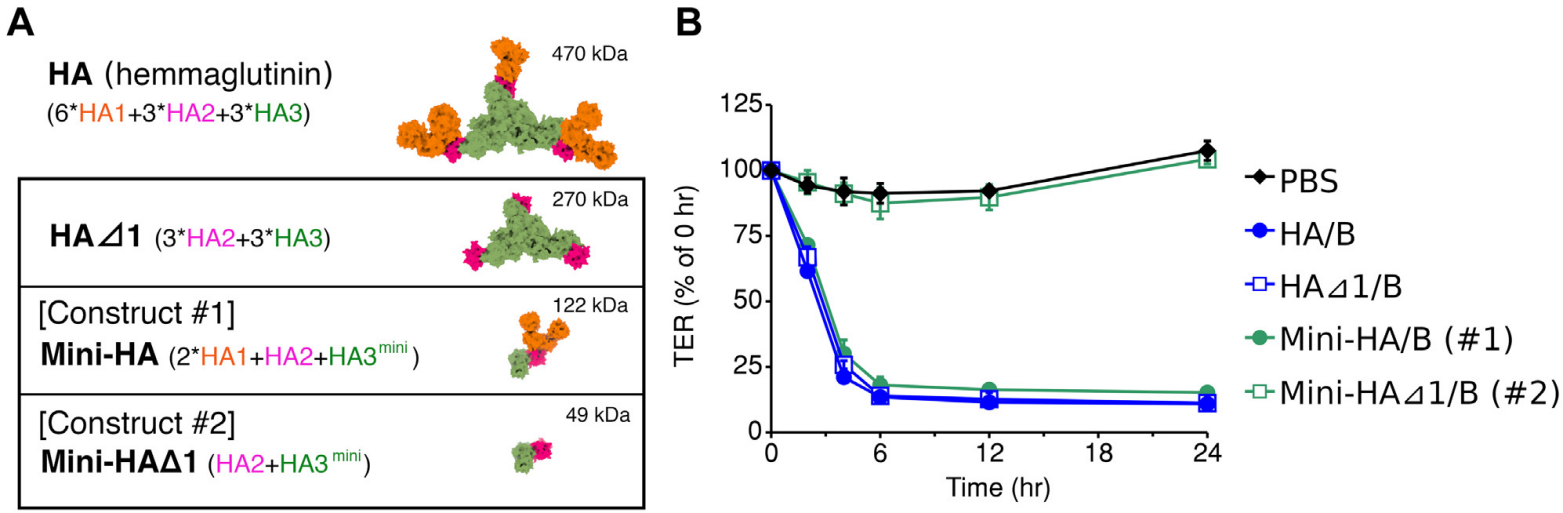
**Barrier-disrupting activities of hemagglutinin (HA)-truncated mutants.***A*, structure of *Clostridium botulinum* HA derived from the serotype B botulinum neurotoxin (BoNT) complex (Protein Data Bank [PDB] ID: 3WIN) and truncated mutants (HAΔ1; Mini-HA, construct #1; Mini-HAΔ1, construct #2). HA1, HA2, and HA3 are represented in *orange*, *magenta*, and *green*, respectively. *B*, transepithelial electrical resistance (TER) of Caco-2 cell monolayers was measured in the presence of 100 protomer nM of HA/B, HAΔ1/B, Mini-HA/B (#1), and Mini-HAΔ1/B (#2) (33.3 nM of HA/B and HAΔ1/B and 100 nM of Mini-HA/B and Mini-HAΔ1/B) at the basolateral sides. Values represent the mean ± SD of triplicate wells.

In the present study, we developed NanoHA, a minimal E-cadherin inhibitor protein derived from HA (termed NanoHA). NanoHA inhibits cell–cell adhesion and disrupts the epithelial barrier, similar to full-length WT HA. Furthermore, NanoHA has one-tenth the molecular weight of full-length HA and can be purified in sufficient quantities using a simple purification strategy.

## Results

### Minimization of HA

**Figure 2:**
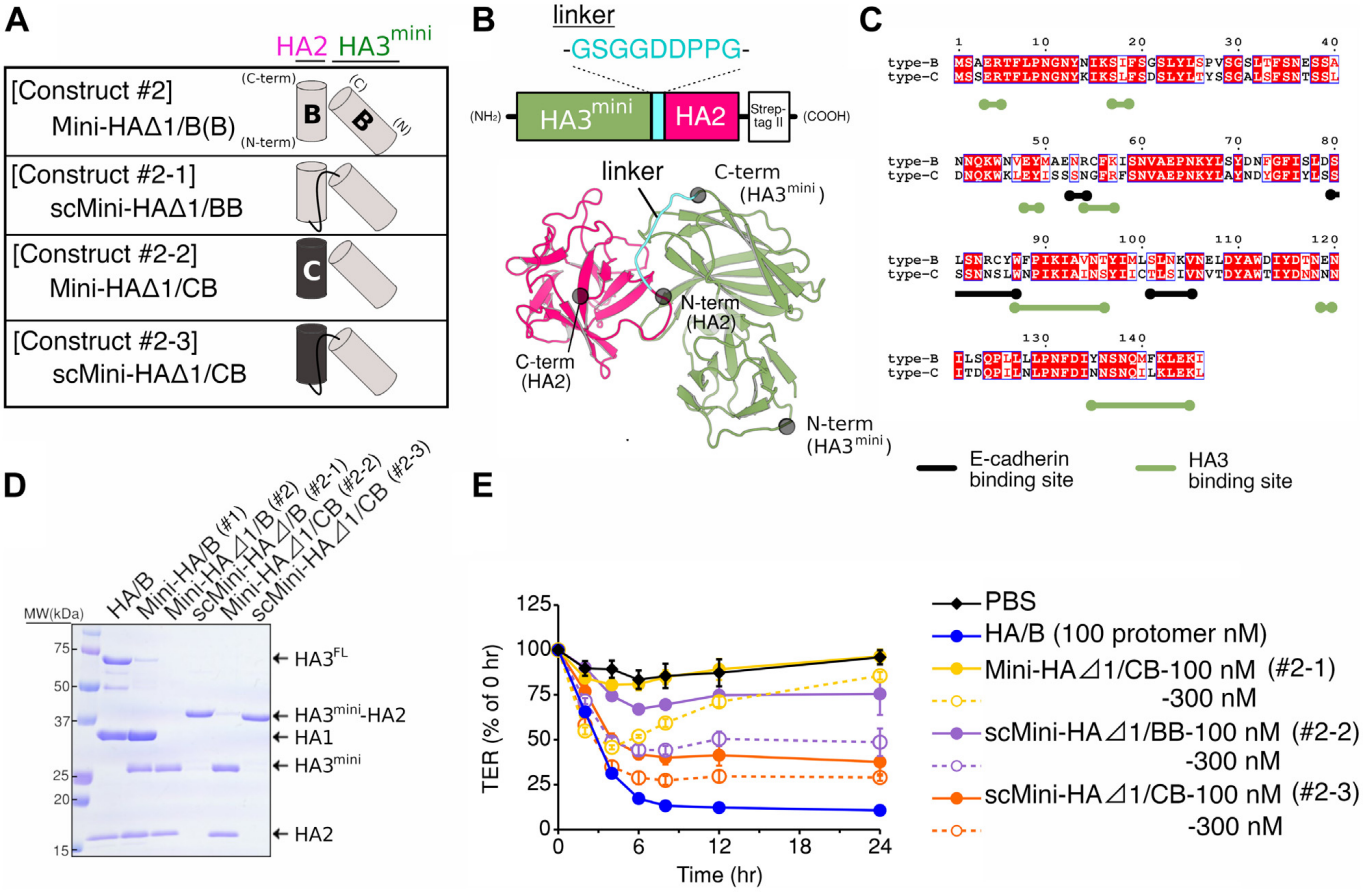
**Molecular engineering of a single-chain CB chimeric Mini-HAΔ1 (scMini-HAΔ1/CB).***A*, schematic models of single-chain (sc) and chimeric Mini-HAΔ1 mutants (#2-1, #2-2, and #2-3). *B*, simulated model of scMini-HA containing a short linker peptide (GSGGDDPPG). *C*, amino acid sequence alignment of HA2/B and HA2/C. *Black and green lines* show the E-cadherin-binding sites and HA3-binding sites of HA2/B, respectively. The figure was generated using ClustalW (39) and the ESPript 3.0 server (40). *D*, SDS-PAGE of HA/B, Mini-HA/B (#1), Mini-HAΔ1/B (#2), scMini-HAΔ1/B (#2-1), Mini-HAΔ1/CB (#2-2), and scMini-HAΔ1/CB (#2-3). The gel was stained with Coomassie brilliant blue (CBB). *E*, transepithelial electrical resistance (TER) of Caco-2 cell monolayers was measured in the presence of 100 or 300 protomer nM of HA/B, scMini-HAΔ1/BB (#2-1), Mini-HAΔ1/CB (#2-2), and scMini-HAΔ1/CB (#2-3) at the basolateral sides. Values represent the mean ± SD of triplicate wells.

To identify the essential HA fragments that disrupt the epithelial barrier, we tested the barrier-disrupting activity of full-length HA/B (HA/B: HA1/B + HA2/B + HA3/B), HA/B lacking HA1/B (HAΔ1/B: HA2/B + HA3/B), HA/B lacking the trimerization domain of HA3/B (Mini-HA/B: HA1/B + HA2/B + HA3^mini^/B, construct #1) (23), and Mini-HA/B lacking HA1/B (Mini-HAΔ1/B: HA2/B + HA3^mini^/B, construct #2) (Figs. 1*A*, 2*D* and S1). Within 4 h post-addition, 100 (protomer) nM HA/B, HAΔ1/B, and Mini-HA/B (#1) reduced the transepithelial electrical resistance (TER) of the Caco-2 cell monolayer to 25% of that at 0 h post-addition, whereas 100 nM of Mini-HAΔ1/B (#2) did not affect the TER (Fig. 1*B*). In this study, we calculated protein concentration in protomer units for HA/B and HAΔ1/B because HA comprises three protomers (3, 23), and each protomer has one E-cadherin-binding site (14): that is, 1 nM HA/B is equal to three protomer nM HA/B.

### Engineering of Mini-HAΔ1

Mini-HA (#1) is observed to bind to E-cadherin with a dissociation constant (*K*_D_) similar to that of HA (∼2.7 μM *versus* ∼2.3 μM) (14) and disrupts the epithelial barrier (Fig. 1*B*) (14, 24). HAΔ1 also binds to E-cadherin and disrupts the epithelial barrier (Fig. 1*B*) (3, 13). These data led us to hypothesize that Mini-HAΔ1 (#2) has potential to disrupt the epithelial barrier, even though Mini-HAΔ1/B failed to do so (Fig. 1*B*). First, we attempted to improve the barrier-disrupting activity of the HA2/B + HA3^mini^/B complex (Mini-HAΔ1, #2). As single-chain (sc) fragment variable fragments are known to be more stable than their fragment variable fragments (25), we designed and tested single-chain Mini-HAΔ1/B (scMini-HAΔ1/B, construct #2-1). Previously, we determined the crystal structure of full-length HA/B (3). To preserve the spatial arrangement between HA2/B and HA3/B, we fused the N terminus of HA2/B and the C terminus of HA3^mini^/B with a short linker sequence (scMini-HAΔ1/B, #2-1). (Figs. 2, *A*, *B* and *D* and S1). We found that 100 and 300 nM of scMini-HAΔ1/B (#2-1) reduced the TER of Caco-2 monolayers to 76% and 49% compared with that at 0 h post-addition, respectively (Fig. 2*E*). However, they did not reduce the TER completely, whereas 100 protomer nM of HA/B did (Fig. 2*E*). These data indicate that fusing HA2 and HA3 *via* a short linker maintains the structural integrity of the natural noncovalent interaction between HA2 and HA3. Next, we replaced HA2/B in Mini-HAΔ1/B with HA2/C (Mini-HAΔ1/CB, construct #2-2) (Figs. 2, *A*, *C* and *D* and S1). HA2/B shows 98% and 64% amino acid sequence homology with HA2/A and HA2/C, respectively. We previously demonstrated that a BCB chimeric full-length HA complex (HA1/B + HA2/C + HA3/B) binds to E-cadherin in a manner similar to HA/B, whereas a BBC chimeric complex (HA1/B + HA2/B + HA3/C) does not (26). Treatment with 100 and 300 nM Mini-HAΔ1/CB (#2-2) reduced TER to 81% and 46% of that at 0 h post-addition, respectively (Fig. 2*E*). These TER values were recovered at 4 h post-addition (Fig. 2*E*). Although chimerization of HA2 does not largely affect the E-cadherin-binding activity itself (26), it may increase barrier-disrupting activity *via* indirect effects such as multimerization (24) or stabilization of the truncated mutant. A single-chain CB chimeric Mini-HAΔ1 (scMini-HAΔ1/CB, construct #2-3) further reduced the TER to 38% at 100 nM and 29% at 300 nM of that at 0 h post-addition (Fig. 2*E*).

### Engineering of NanoHA

**Figure 3:**
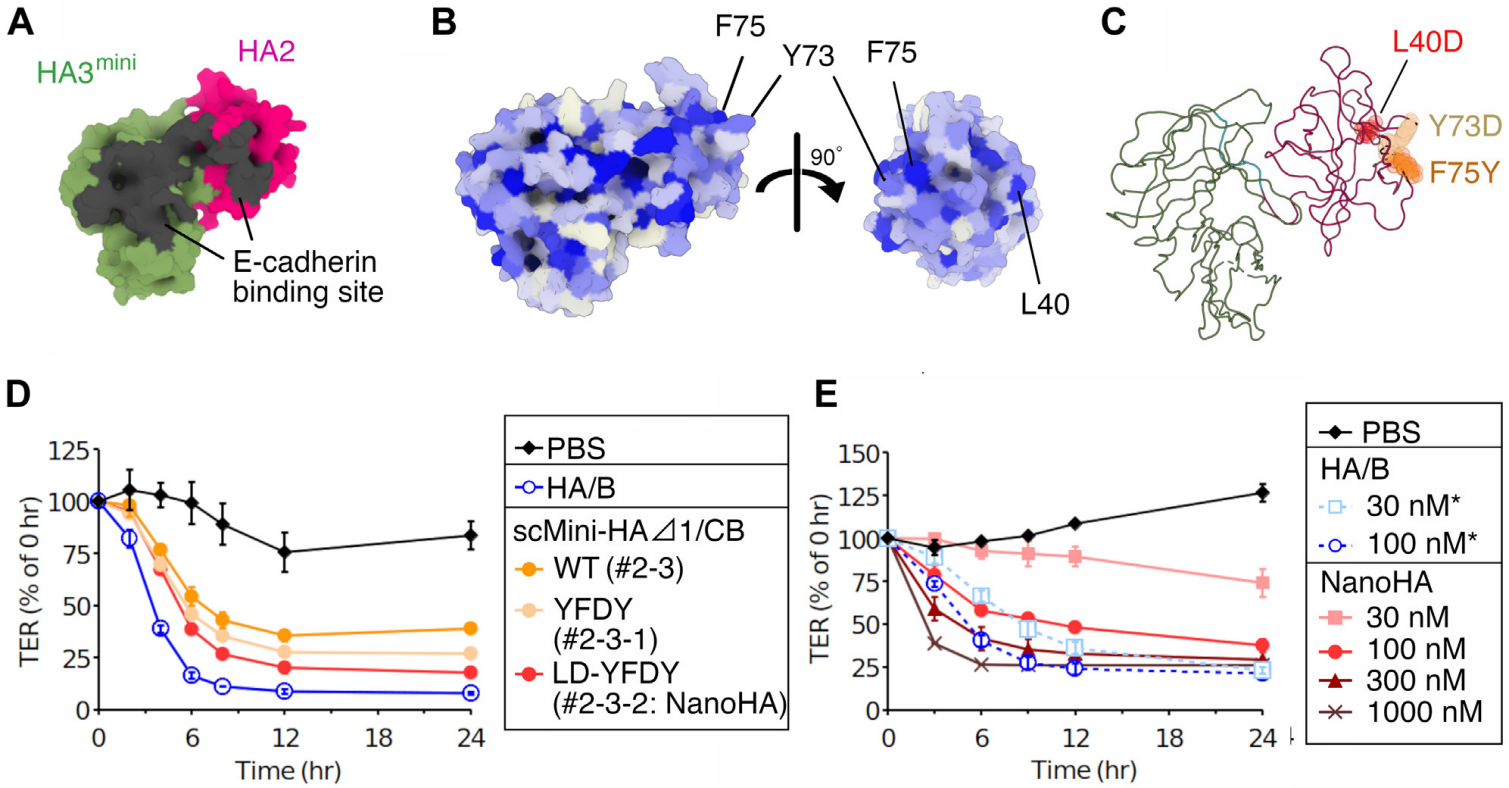
**Molecular engineering of scMini-HAΔ1/CB-LD-YFDY (NanoHA).***A*, E-cadherin-binding sites are represented as *gray areas* on the surface model of Mini-HAΔ1/B (#2). HA2 and HA3^mini^ are represented in *magenta* and *green*, respectively. *B*, surface hydrophobicity of Mini-HAΔ1/CB. The color of the surface represents the hydrophobicity level. *White*, *light blue*, and *blue* represent low, medium, and high hydrophobicity, respectively. *C*, Leu40, Tyr73, and Phe75 of HA2 are represented as *spheres* on a wire model of Mini-HAΔ1/CB. *D* and *E*, TER of the Caco-2 cell monolayers was measured in the presence of 100 protomer nM of HA/B and scMini-HAΔ1/CB (WT, #2-3; YFDY, #2-3-1; LD-YFDY, #2-3-2) (*D*) or in the presence of HA/B (30 or 100 protomer nM; *asterisks* indicate protomer nM) and scMini-HAΔ1/CB-LD-YFDY (#2-3-2; NanoHA; 30, 100, 300, or 1000 nM) (*E*) at the basolateral side. Values represent the mean ± SD of triplicate wells. HA, hemagglutinin.

Hydrophobic to hydrophilic amino acid mutations on the protein surface improve protein solubility (27). To further improve the barrier-disrupting activity, we introduced amino acid substitutions in the surface region of scMini-HAΔ1/CB, except for the E-cadherin-binding sites, to decrease hydrophobicity (Figs. 3, *A–C* and S1). We found that scMini-HAΔ1/CB with Tyr73Asp and Phe75Tyr mutations in HA2/C (YFDY, construct #2-3-1) decreased the TER of the Caco-2 cell monolayer more effectively than WT scMini-HAΔ1/CB (27% *versus* 39%) (Fig. 3*D*). Furthermore, scMini-HAΔ1/CB-YFDY with a Leu40Asp mutation in HA2/C (LD-YFDY, construct #2-3-2) decreased TER more effectively than did the YFDY mutant (18% *versus* 27%) (Fig. 3*D*). We found that 100 and 300 nM of scMini-HAΔ1/CB-LD-YFDY (#2-3-2, termed NanoHA) exhibited barrier-disrupting activity similar to that of 30 protomer nM and 100 protomer nM of HA/B, respectively (Fig. 3*E*). These results indicate that the HA2 + HA3^mini^ complex is the intrinsic core component essential for HA to inhibit E-cadherin and disrupt the epithelial barrier.

### Binding affinity and kinetics for E-cadherin

**Figure 4:**
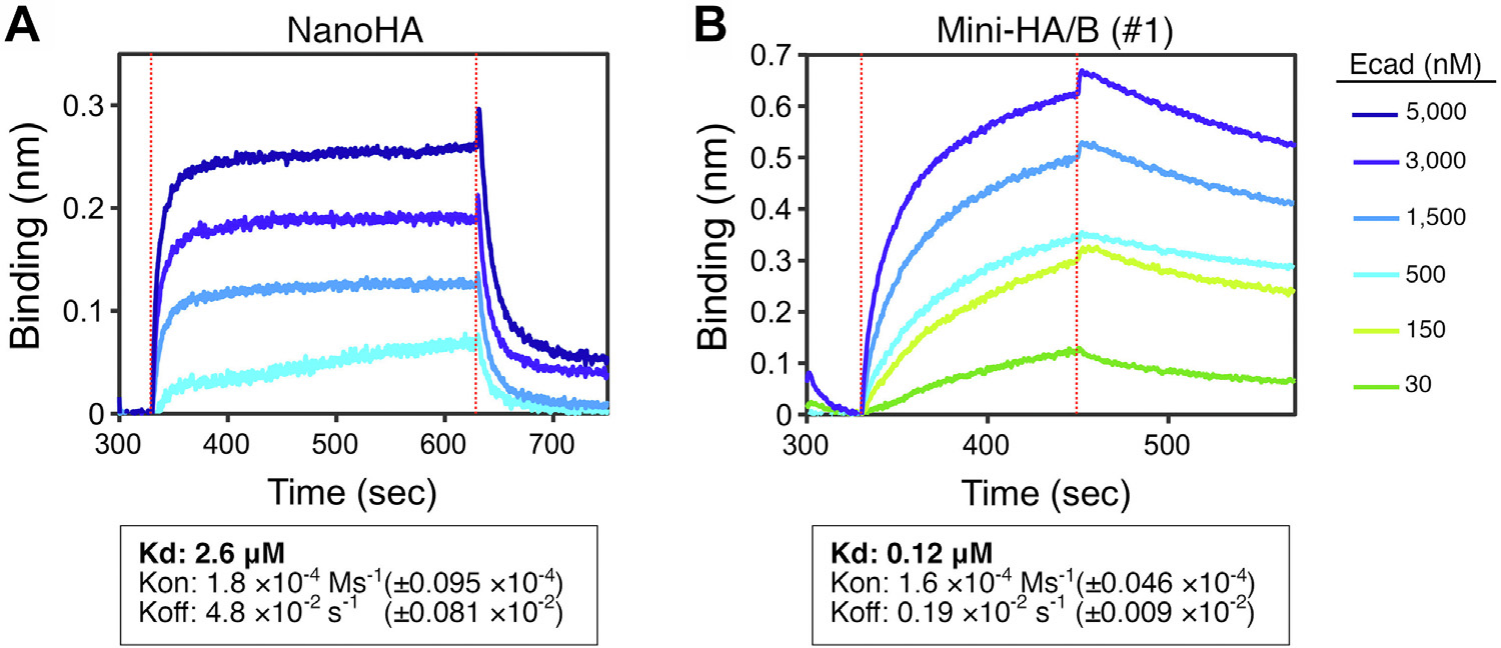
**Binding affinity and kinetics of NanoHA for E-cadherin.** Biolayer interferometry (BLI) assay was performed using a BLItz system. AMC biosensors were pre-immobilized with an anti-*Strep*-tag II tag antibody and then coated with *Strep*-tag II-tagged NanoHA (*A*) or Mini-HA/B (#1; *B*). The BLI sensorgrams were obtained using E-cadherin EC1–5 (Ecad, 30–5000 nM). Association and dissociation rate constants (*K*_on_ and *K*_off_) and dissociation constant (*K*_D_) were determined by global fitting to a 1:1 binding model. *Red dotted lines* indicate the start of the association (*left*) and dissociation (*right*) phases. HA, hemagglutinin.

NanoHA binds to E-cadherin with lower affinity (*K*_D_ ∼2.6 μM) than Mini-HA/B (#1; *K*_D_ ∼0.12 μM) (Fig. 4). The association rate constant (*K*_on_) is similar between NanoHA (1.8 × 10^−4^ Ms^−1^) and Mini-HA/B (1.6 × 10^−4^ Ms^−1^), whereas NanoHA has a faster dissociation rate constant (*K*_off_) than Mini-HA/B (4.8 × 10^−2^ s^−1^ *versus* 0.19 × 10^−^^2^ s^−1^). Thus, the difference in binding affinity (*K*_D_) is attributed to a faster *K*_off_ of NanoHA. These results suggest that the fast *K*_off_ affects the barrier-disrupting activity of NanoHA.

### Inhibition of cell–cell adhesion

**Figure 5:**
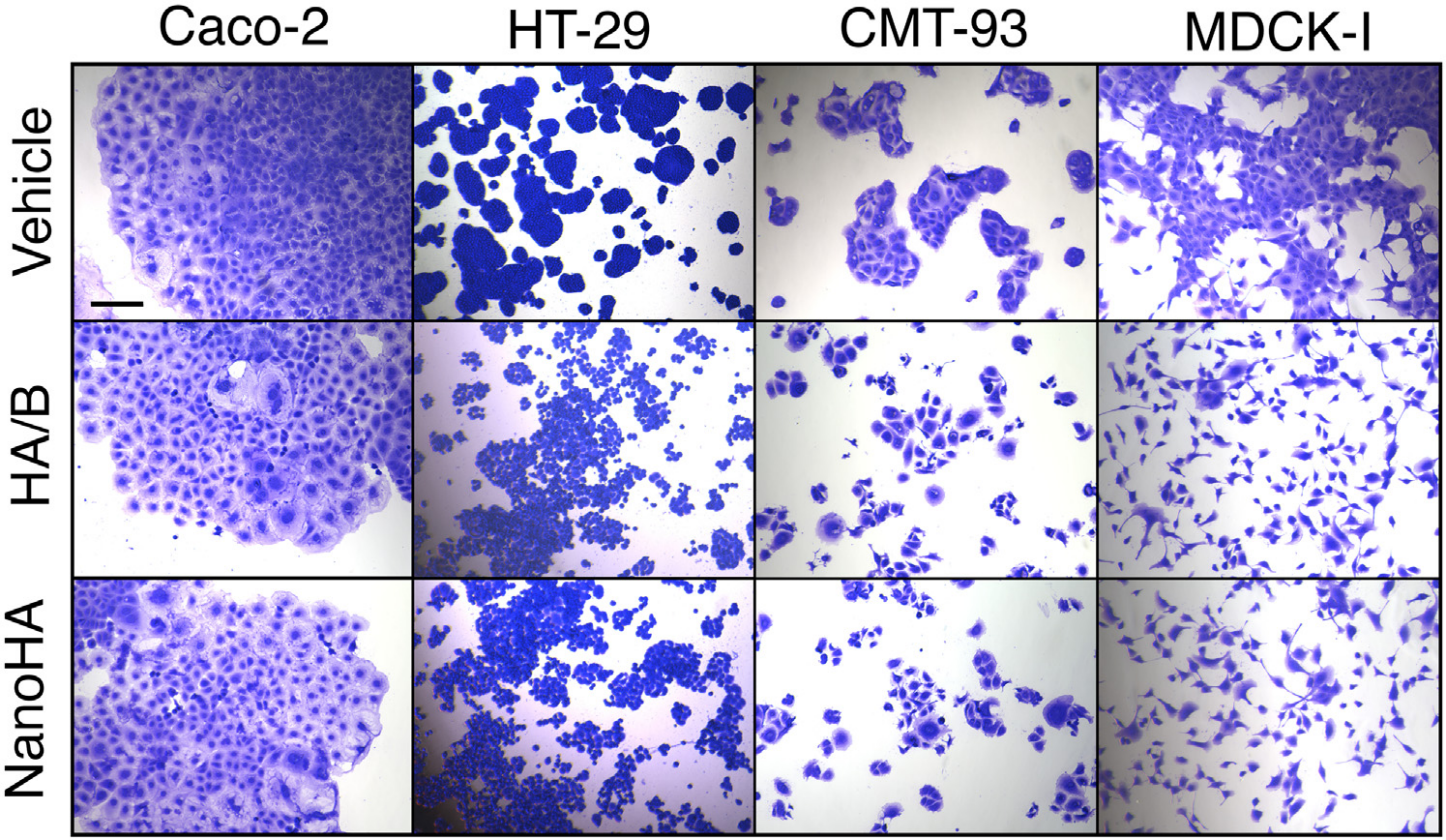
**NanoHA inhibits the cell–cell adhesion of epithelial cells.** Caco-2, HT-29, CMT-93, and MDCK-I cells were cultured with vehicle (PBS), 30 nM (90 protomer nM) HA/B, or 100 nM NanoHA for 24 h and stained with Giemsa stain solution. The scale bar represents 200 μm. HA, hemagglutinin; MDCK, Madin–Darby canine kidney.

To test the effect of NanoHA on cell–cell adhesion in different cell lines, we added NanoHA to Caco-2, HT-29 (human colon carcinoma–derived epithelial cell line), CMT-93 (mouse rectum carcinoma–derived epithelial cell line), and MDCK-I cells. We found that NanoHA inhibited cell–cell adhesion similarly to HA/B (Figs. 5 and S2). In particular, the colonies of HT-29, CMT-93, and MDCK-I cells were dispersed by these treatments (Figs. 5 and S2). The treated cells largely remained attached to the culture plates (Figs. 5 and S2), suggesting that HA and NanoHA did not inhibit cell–matrix adhesion.

### Binding to the basolateral cell surface at 4 °C

**Figure 6:**
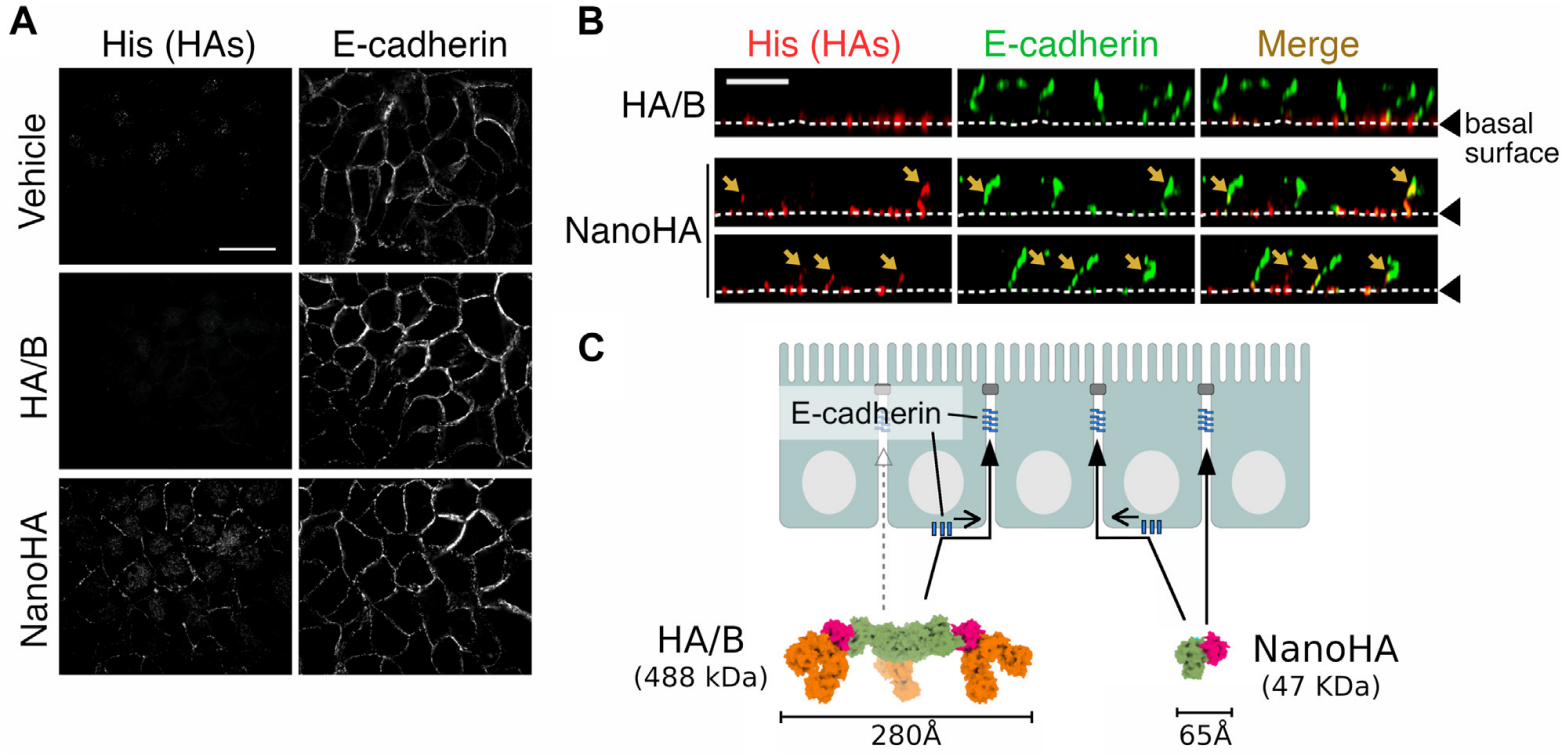
**Binding of HAs in the lateral intercellular space at 4 °C.***A* and *B*, Caco-2 cell monolayers were incubated with His-tagged HA/B or NanoHA applied to basolateral chambers at 4 °C for 40 min. The cells were stained with anti-His-tag and anti-E-cadherin antibodies. The images show the XY planes (*A*) and XZ planes (*B*). *Arrows* show HAs that reside on the lateral cell surface. *White dotted lines* show the basal cell surface. The scale bars represent 20 μm (*A*) and 10 μm (*B*). *C*, proposed model of HA accessibility to the lateral intercellular space. HA/B binds to the basal surface, but not to the lateral surface, of cells at 4 °C and is transported from the basal surface to the lateral surface at 37 °C (24). Meanwhile, NanoHA binds to both surfaces at 4 °C, as shown in (*A* and *B*). HA, hemagglutinin.

To approach AJs from the basolateral side, basolaterally applied HA/B initially binds to the basal surface of Caco-2 cells. The cell-bound HA/B is then transported to the lateral surface of the cell in an E-cadherin-binding ability–dependent manner (24). We propose that cadherin flow (28) transports HA from the basal to lateral surface. NanoHA comprises the core components of HA, which are essential to inhibit E-cadherin and has one-tenth molecular weight (47 kDa *versus* 488 kDa) and one-fourth molecular size (65 Å *versus* 280 Å diameter) compared with that of full-length HA (Figs. 1*A* and 6*C*). Therefore, we hypothesized that NanoHA is advantageous in terms of accessibility to intercellular spaces. To test this hypothesis, we added HAs to the basolateral sides of Caco-2 cell monolayers and incubated them at 4 °C. Consequently, NanoHA was localized to both the basal and lateral cell surfaces (Fig. 6, *A* and *B*), whereas HA/B bound only to the basal surface (Fig. 6, *A* and *B*) (24). This indicates that NanoHA can access AJs by passive diffusion, and that HA/B is too large to penetrate lateral intercellular spaces (Fig. 6*C*).

## Discussion

In this study, we developed a minimal E-cadherin inhibitor protein, NanoHA, derived from *C. botulinum* HA through rational design. scMini-HAΔ1/CB-LD-YFDY (construct #2-3-2, termed NanoHA) has three types of modifications: a short peptide linker to preserve structural integrity, chimerization, and three point mutations (Figs. 2*B*, 3, *B* and *C* and S1). NanoHA inhibited cell–cell contacts and disrupted the epithelial barrier (Figs. 3*E*, 5 and S2). To obtain high-quality purified recombinant protein, full-length HA needs four purification steps: purification of HA1, HA2, and HA3 and full-length HA after *in vitro* reconstitution. In contrast, NanoHA can be purified in a single step and shows a 10-fold higher protein yield than that of full-length HA (data not shown).

Cadherin-mediated cell–cell adhesions are abrogated by other cadherin inhibitors, such as ADH-1 (also known as Exherin), a cyclic peptide composed of the His-Ala-Val sequence of N-cadherin (29); Epep, an E-cadherin mimic peptide H-SWELYYPLRANL-NH2 (30); and E-cadherin-neutralizing antibodies (31, 32). These E-cadherin inhibitors induce apoptosis in some cell lines by abrogating cell–cell contact (32, 33, 34). In contrast, HA/A and HA/B are not toxic to epithelial cells, such as Caco-2 cells and MDCK-I cells, but rather promote cell proliferation (19). NanoHA also does not affect cell viability of Caco-2, HT-29, CMT-93, MDCK, and HeLa cells (Fig. S3). Recently, we reported a simple and robust method to maintain iPS cells in an undifferentiated state using full-length HA (20, 21, 22). We demonstrated that NanoHA, as well as full-length HA, disrupts the epithelial barrier (Fig. 3*E*) and inhibits E-cadherin-mediated cell–cell contacts in epithelial cells (Figs. 5 and S2). Thus, NanoHA can be used in novel iPSC culture systems. NanoHA (47 kDa) has one-tenth the molecular weight of full-length HA (native HA, 470 kDa; recombinant HA including affinity tags, 488 kDa) (Fig. 1*A*) and is favorable for penetrating intercellular spaces (Fig. 6). Therefore, NanoHA has the advantage of being an E-cadherin inhibitor for tightly packed 3D-cultured iPSC aggregates compared with full-length HA. Furthermore, NanoHA provides a wide variety of applications as a useful basal protein tool for further molecular engineering, such as directed evolution using phage display (35), and addition of other protein components (36) or functional signal peptides such as cell-targeting peptides (37).

## Experimental procedures

### Plasmid construction

Genomic DNA was extracted and purified from *C. botulinum* serotype B strain Okra and serotype C strain Stockholm. HA1 (amin acids 7–294) derived from the serotype B BoNT complex (HA1/B)-encoding gene, excluding the stop codon, was cloned into the NheI–SalI site of the pET28b(+) vector (Merck), and an oligonucleotide encoding a FLAG tag was inserted at the C terminus of HA1/B. HA2 (amino acids 2–146) derived from the serotype B BoNT complex (HA2/B) or serotype C BoNT complex (HA2/C) encoding gene was cloned into the NheI–SalI site of the pET28b(+) vector. Full-length HA3 (amino acids 19–626) derived from the serotype B BoNT complex (HA3/B)-encoding gene, excluding the stop codon, was cloned into the NcoI–SalI site of the pET52b(+) vector (Merck). The truncated mutant of HA3 (termed HA3^mini^, amino acids 380–626) derived from the serotype B BoNT complex (HA3^mini^/B)-encoding gene, excluding the stop codon, was cloned into the NcoI–SalI site of the pET52b(+) vector. An oligonucleotide encoding a *Strep*-tag II tag was inserted at the C termini of HA3 and HA3^mini^. For single-chain Mini-HAΔ1 (scMini-HAΔ1) proteins, HA3^mini^, an oligonucleotide encoding a short linker sequence (GSGGDDPPG), HA2, and an oligonucleotide encoding a *Strep*-tag II tag were cloned into the NcoI–SalI site of the pET52b(+) vector. Site-directed mutagenesis was performed using PrimeSTAR Max Polymerase (TaKaRa Bio). The inserted regions of these plasmids and the presence of mutations were confirmed by DNA sequencing.

### Protein expression and purification

Rosetta2 (DE3) *Escherichia coli* cells (Merck) were grown in Terrific broth medium. The expression of HA proteins was induced using Overnight Express Autoinduction System 1 (Merck) at 18 °C for 48 h. Cells were harvested and lysed in PBS (pH 7.4) by sonication. His-HA1-FLAG, His-HA2, and His-NanoHA were purified using HisTrap HP (Cytiva). *Strep*-tag II-tagged proteins were purified using StrepTrap HP (Cytiva). These column purification steps were performed following the manufacturer’s protocols. All proteins were dialyzed against PBS (pH 7.4) and stored at −80 °C until further analysis. The protein concentration of the samples was determined using the Pierce bicinchoninic acid assay (Thermo Fisher Scientific).

### *In vitro* reconstitution and purification

The HA protein complexes were reconstituted and purified as previously described (3). For HA and Mini-HA, purified HA1, HA2, and HA3 (or HA3^mini^) were mixed at a molar ratio of 4:4:1. For HAΔ1 and Mini-HAΔ1, purified HA2 and HA3 (or HA3^mini^) were mixed at a molar ratio of 4:1.

### TER assay

TER was measured using Millicell-ERS (Merck) as previously described (12). Briefly, HAs were added to the basolateral chambers of a Transwell (Corning) with the Caco-2 cell monolayer, and the plates were incubated at 37 °C with 5% CO_2_. TER was measured at time points up to 24 or 72 h postaddition.

### Optical microscopy

Caco-2, HT-29, CMT-93, and MDCK cells were incubated with 0 to 100 (protomer) nM of HA/B or NanoHA at 37 °C with 5% CO_2_ for 24 h in 24-well plates (Iwaki). The plates were stained with Giemsa Stain Solution (FUJIFILM Wako Chemicals) according to the manufacturer’s protocol and observed using a BZ-X700 all-in-one fluorescence microscope (KEYENCE).

### Immunofluorescence

The basolateral side of Caco-2 cells grown on a Transwell was treated with 17 nM (51 protomer nM) of HA/B (His-HA1/B-FLAG + His-HA2/B + HA3/B-strep), or 51 nM of His-NanoHA were added to the basolateral chamber of Transwell with the Caco-2 cell monolayer. The plates were then incubated at 4 °C for 40 min. After washing, the cells on Transwell filter membranes were fixed with 4% PFA at room temperature for 15 min, permeabilized with 0.5% Triton X-100/PBS at room temperature for 5 min, and blocked with 5% bovine serum albumin/PBS. The cells were then incubated with mouse anti-His-tag monoclonal antibody (OGHis, MBL; 1:1000 dilution) and rat anti-E-cadherin monoclonal antibody (DECMA-1, Merck; 1:1000 dilution), followed by Alexa Fluor 488-conjugated anti-mouse immunoglobulin G antibody (Thermo Fisher Scientific; 1:400 dilution) and Cy3-conjugated anti-rat immunoglobulin G antibody (Jackson ImmunoResearch; 1:400 dilution). The slides were mounted with ProLong Diamond Antifade mountant. Images were acquired by confocal microscopy using an IX71 microscope (Olympus) and a CSUX1 confocal scanner unit (Yokogawa Electric) and processed using Metamorph software (Molecular Devices).

### Structure modeling of NanoHA

The molecular model of MiniHAΔ1/CB was built by manually docking the crystal structures of HA2 from the HA1/D–HA2/D complex (Protein Data Bank ID: 2E4M) onto that of HA/B (Protein Data Bank ID: 3WIN), as HA2/D shows 99% amino acid sequence homology with HA2/C. A short peptide linker sequence, Gly-Ser-Gly-Gly-Asp-Asp-Pro-Pro-Gly, was inserted between the C terminus of HA3^mini^/B and the N terminus of HA2/D, and the model was refined by simulated annealing using GROMACS (GROMACS Development Team) (38). The figures were generated using PyMOL (The PyMOL Molecular Graphics System, version 2.4.0, Schrödinger, LLC).

### Biolayer interferometry analysis

Dissociation constant (*K*_D_), association rate constant (*K*_on_), and dissociation rate constant (*K*_off_) were measured on a BLItz system (ForteBio). About 1 μM *Strep*-tag II-tagged NanoHA or Mini-HA/B (#1) were immobilized on Octet AMC Biosensors (SARTORIUS) coated with 100 μg/ml Anti-*Strep*-tag II tag monoclonal antibody (StrepMAB-Immuno; IBA GmbH). About 30 to 5000 nM of mouse E-cadherin EC1–5 purified as described previously (13) were then loaded onto the biosensors equilibrated with kinetics buffer (20 mM Tris [pH 7.4], 100 mM NaCl, 2 mM CaCl_2_, 0.1% bovine serum albumin, and 0.01% Tween-20). The data were analyzed by global fitting to a 1:1 binding model using BLItz Pro software (ForteBio; version 1.2).

### Cell proliferation assays

Caco-2, HT-29, CMT-93, MDCK, and HeLa (human cervical carcinoma–derived epithelial-like cell line) cells were seeded at 1 × 10^4^ cells/well in 96-well plates (Iwaki) and cultured for 24 h in Dulbecco’s modified Eagle’s medium (Thermo Fisher Scientific) supplemented with 20% (for Caco-2 cells) or 10% (for HT-29, CMT-93, MDCK, and HeLa cells) fetal bovine serum. The cells were further cultured with 1000 protomer nM HAs or 1% Triton X-100 at 37 °C for 24 h. Cell viability was examined using the cell proliferation reagent Cell Count Reagent SF (Nacalai Tesque) following the manufacturer’s protocols.

## Data availability

All experimental data are contained within the article.

## Supporting information

This article contains supporting information.

## Conflict of interests

The authors declare that they have no conflicts of interest with the contents of this article.
