# Supplementary material for: Molecular engineering of a minimal E-cadherin inhibitor protein derived from Clostridium botulinum hemagglutinin
Source: J Biol Chem. 2023 Jan 25;299(3):102944. doi: 10.1016/j.jbc.2023.102944 (PMC9958082; doi:10.1016/j.jbc.2023.102944)
Supplement: Supplemental Figures S1–S3 [file mmc1.docx]

**Supporting Information**

Molecular engineering of a minimal E-cadherin inhibitor protein derived from *Clostridium botulinum* hemagglutinin

Sho Amatsu^1,2*^, Takuhiro Matsumura^1^, Masahiko Zuka^2^, and Yukako Fujinaga^1*^

^1^Department of Bacteriology, Graduate School of Medical Sciences, Kanazawa University, Ishikawa, Japan

^2^Department of Forensic Medicine and Pathology, Graduate School of Medical Sciences, Kanazawa University, Ishikawa, Japan

Running title: Engineering of E-cadherin inhibitor protein

* For correspondence: Sho Amatsu, [amatsu@med.kanazawa-u.ac.jp](mailto:amatsu@med.kanazawa-u.ac.jp); Yukako Fujinaga, [yukafuji@med.kanazawa-u.ac.jp](mailto:yukafuji@med.kanazawa-u.ac.jp)


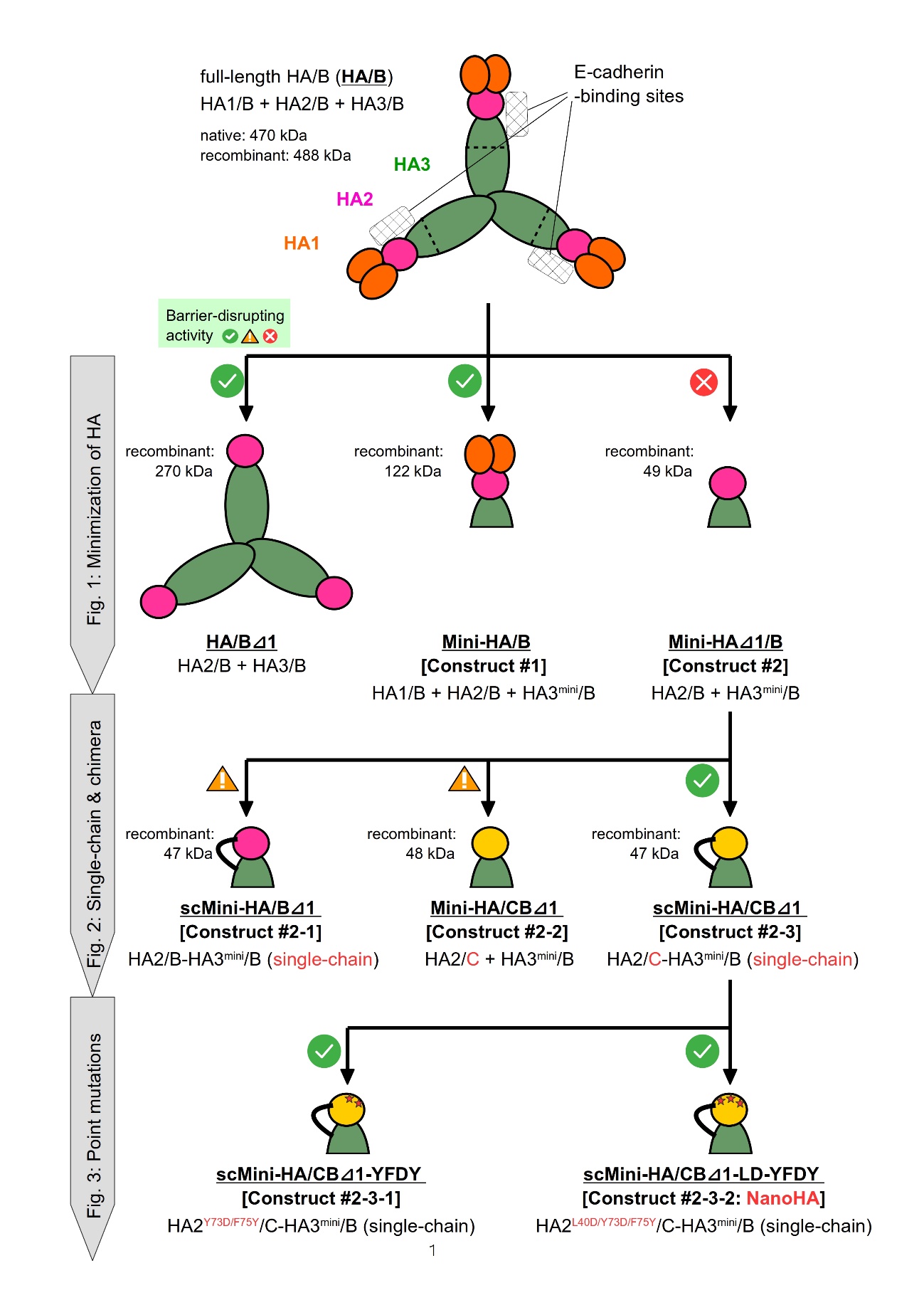


##### Figure S1. A schematic diagram illustrating the protein engineering of NanoHA


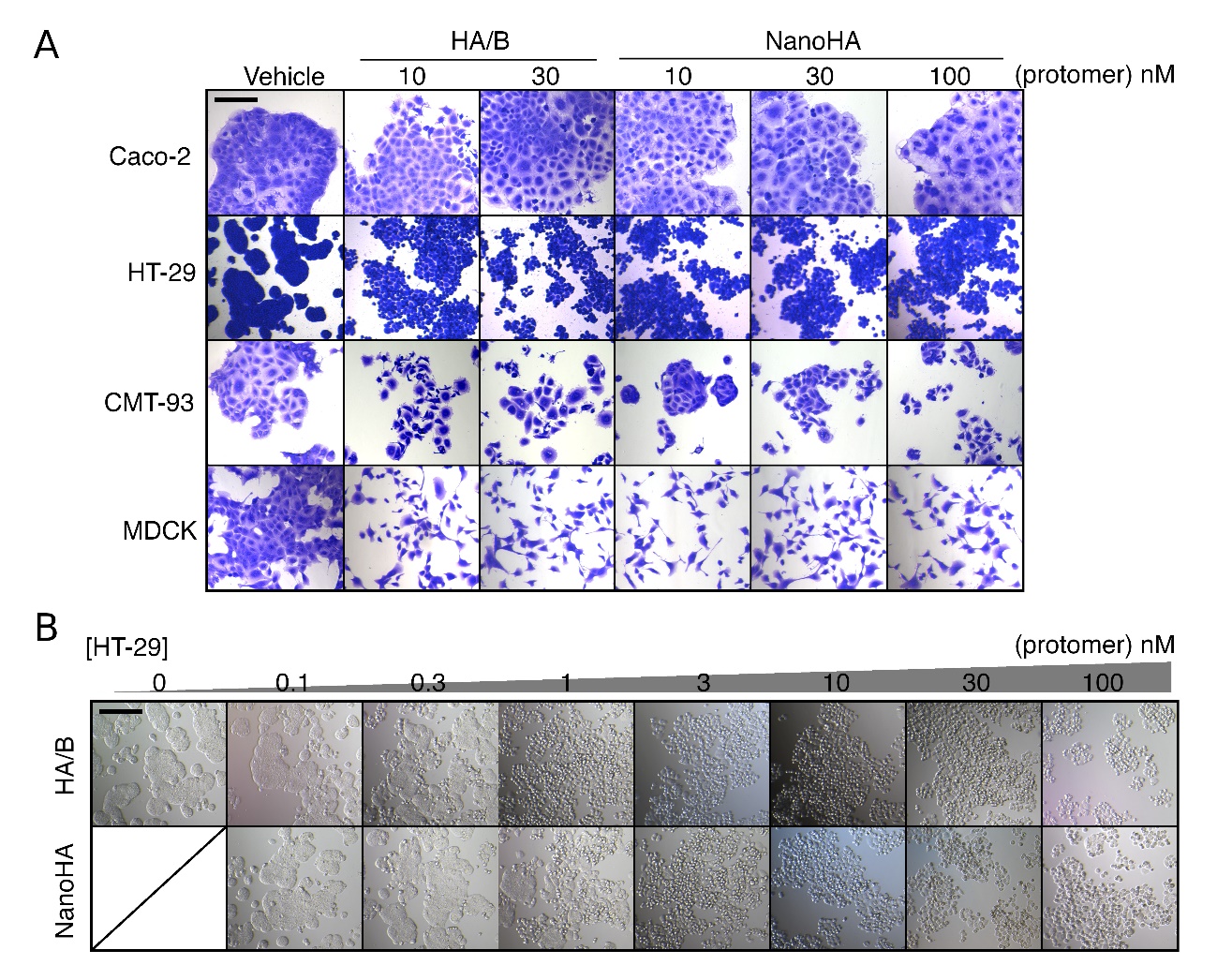


##### Figure S2. Cell-cell contact inhibition of NanoHA

*A*, Caco-2, HT-29, CMT-93, and MDCK cells were cultured with 10 and 30 protomer nM HA/B or 10, 30, and 100 nM NanoHA for 24 hr, and then stained with Giemsa Stain Solution. Notably, the cell-cell adhesion of CMT-93 cells was inhibited by 10 protomer nM HA/B and 30 nM NanoHA. That of HT-29 and MDCK cells was inhibited by at least 10 (protomer) nM HA/B and NanoHA. The figures showing the vehicle (HT-29, MDCK), the 30 protomer nM HA/B (CMT-93), and the 100 nM NanoHA (HT-29, CMT-93, MDCK) have been derived from same image shown in Fig. 5. Scale bar: 200 μm.

*B*, HT-29 cells were cultured with 0.1–100 (protomer) nM HA/B and NanoHA for 24 hr. The cell-cell adhesion of HT-29 cells was inhibited by 0.3 protomer nM HA/B and 1 nM NanoHA. Scale bar: 200 μm.


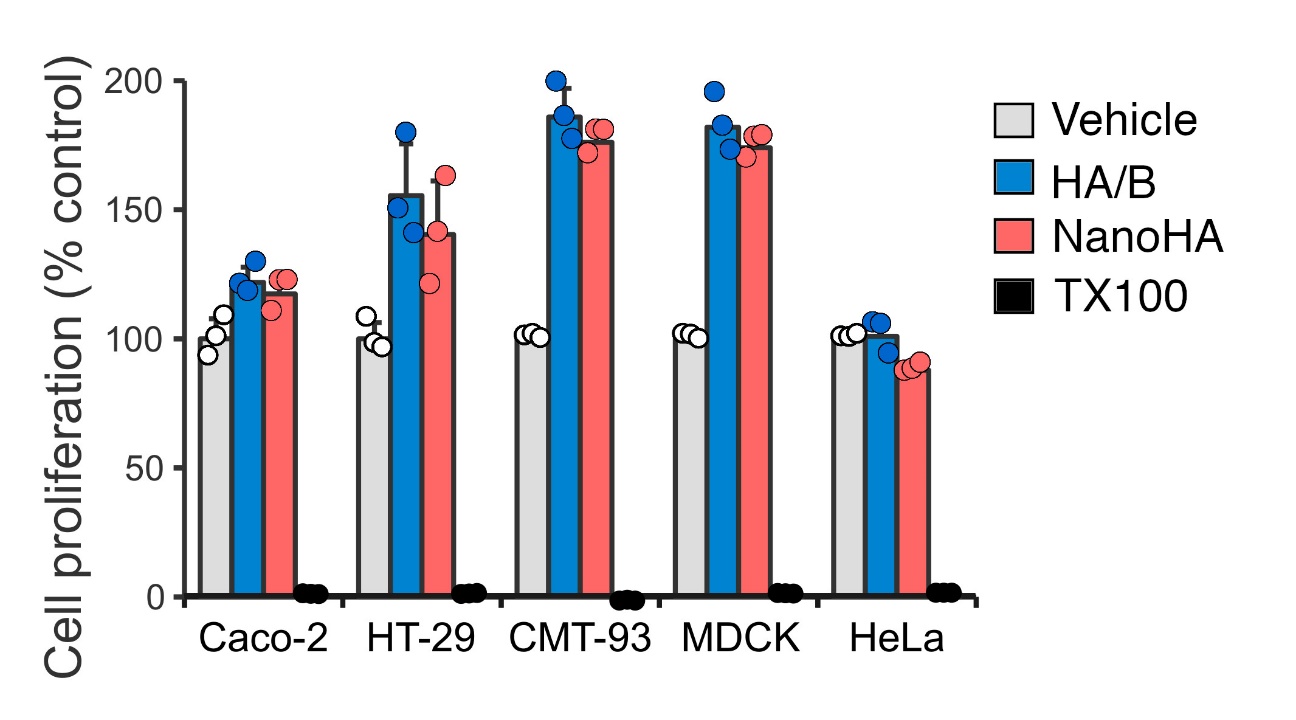


##### Figure S3. Cell proliferation assay

Caco-2, HT-29, CMT-93, MDCK, and HeLa cells were cultured with vehicle (PBS), HA/B (1000 protomer nM), NanoHA (1000 nM), or Triton X-100 (TX100, 1%) for 24 hr. The cell proliferation was evaluated by tetrazolium salt colorimetric method. HA/B and NanoHA promoted the cell proliferation of the cell lines which form E-cadherin-based adhesion (Caco-2, HT-29, CMT-93, MDCK), and did not affect the cell proliferation of the cell line which forms N-cadherin-based adhesion (HeLa). Bars represent the mean (bar) ± SD of triplicate wells; dots represent each data point.
